# Aspirin using was associated with slower cognitive decline in patients with Alzheimer’s disease

**DOI:** 10.1371/journal.pone.0252969

**Published:** 2021-06-16

**Authors:** Jinyan Weng, Guanan Zhao, Liyan Weng, Jingjing Guan, for the Alzheimer’s Disease Neuroimaging Initiative

**Affiliations:** 1 Department of Pharmacy, The Central Hospital of Lishui City, Lishui, Zhejiang, China; 2 Department of Urology, Lishui City People’s Hospital, Lishui, Zhejiang, China; 3 Department of Pharmacy, Lishui City People’s Hospital, Lishui, Zhejiang, China; Ehime University Graduate School of Medicine, JAPAN

## Abstract

We aimed to examine whether the use of aspirin is associated with change in cognitive performance over time, and whether this association is modified by the cognitive stages. This study included a total of 1866 subjects, including 509 subjects with normal cognition (NC), 985 subjects with mild cognitive impairment (MCI), and 372 patients with Alzheimer’s disease (AD). In each group, we further categorized our subjects into two groups based on their aspirin using conditions: Aspirin users and non-aspirin users. Mini-Mental State Examination (MMSE) was the cognitive outcome. Linear mixed models were conducted to examine the longitudinal relationship between the use of aspirin and cognitive performance in each diagnostic group. In the cross-sectional analysis, there were no significant differences in MMSE scores between non-aspirin users and aspirin users in subjects with NC, subjects with MCI or patients with AD. In the longitudinal analysis, we detected an association of the baseline use of aspirin with cognitive decline (MMSE) over time in patients with AD, but not in the NC group or MCI group. Specifically, in AD patients, the use of aspirin at baseline was associated with slower cognitive decline over time. Our data may support an association between the use of aspirin and slower cognitive decline, while this association may be dependent on the clinical stages.

## Introduction

Alzheimer’s disease (AD) is a neurodegenerative disorder characterized by cognitive impairment, hippocampal atrophy, and accumulations of several pathological markers, such as extracellular senile plagues and intracellular neurofibrillary tangles [1].

However, the pathogenesis of AD is not fully clarified and remains a topic of debate. Emerging evidence has suggested that neuroinflammation may play a critical role in the progression of AD and cognitive decline [2]. Regarding the strong relationship between neuroinflammation and AD, it’s reasonable to propose that some anti-inflammatory agents, such as aspirin, may be able to slow down the development of AD and cognitive decline [3]. For example, many previous investigations have suggested that the use of non-steroidal anti-inflammatory drugs (NSAIDs) or aspirin was associated with a lower risk of developing AD [3–10]. However, several studies did not support this beneficial effect of aspirin or NSAIDs on AD [11–13]. In addition, several clinical trials did not find a significant effect of aspirin on reducing risk of dementia or cognitive decline among older people [14, 15]. In general, existing data on the association of aspirin with cognitive decline are inconsistent.

In the present study, we examined the cross-sectional and longitudinal relationships between the use of aspirin and cognitive decline. Further, we also tested the question that whether this association was modified by the different clinical stages. Linear mixed models were used to examine the association of the use of aspirin with cognitive decline over time within the whole sample and within each diagnostic group (subjects with normal cognition, subjects with mild cognitive impairment, and patients with AD).

## Materials and methods

### Data source

The data used in our study were extracted from the Alzheimer’s Disease Neuroimaging Initiative (ADNI) database. Since 2003, the ADNI study has been launched by the National Institute on Aging (NIA), the Food and Drug Administration (FDA), the National Institute of Biomedical Imaging and Bioengineering (NIBIB), non-profit organizations and private pharmaceutical companies. The detailed information about this dataset can be found at the website: http://adni.loni.usc.edu/. In brief, the primary aim of ADNI has been to examine the progression of cognitive symptoms among several groups of participants, including subjects with normal cognition (NC), subjects with mild cognitive impairment (MCI) and patients with Alzheimer’s disease (AD). The ADNI database contains a variety of variables, such as demographical data, cognitive assessments, medical history, neuroimaging measures and other fluid markers. All subjects were requested to provide written informed consent, and the ADNI study was approved by local institutional review board in every ADNI site.

### Participants

In the present study, we included a total of 1866 subjects, including 509 subjects with NC, 985 subjects with MCI, and 372 patients with AD. In each group, we further categorized our subjects into two groups based on their aspirin using conditions: Aspirin users and non-aspirin users (Table 1). With regard to the amount of aspirin usage, 605 aspirin users (81.9%) took 81 mg per day, while 134 aspirin users took aspirin with a variety of dosages (e.g. 325, 85, 160, 80, 162 and 800 mg daily). The dosage of aspirin used by individuals with normal cognition, MCI and AD patients were 123.7 ± 91 (mean ± SD), 113.5 ± 88 and 130 ± 98 mg daily, respectively. One MCI patient and one AD patient did not report the exact dosage of aspirin. Therefore, they were excluded from the calculation of the means and SDs of the dosage of aspirin. However, they were still included in other analyses. The baseline aspirin using status (Aspirin users vs non-aspirin users) was treated as a predictor in our linear mixed-effects models. The authors cannot access to information that could identify participants.

**Table 1:** Characteristics of three cognitive groups as a function of Aspirin using status.

	Aspirin using status		
Characteristics	Non-aspirin users	Aspirin users	P values
NC (n = 509)			
Sample size, n	310	199	/
Age, years	73.4 ± 6,14	74.4 ± 5.89	0.08
Education, years	16.4 ± 2.72	16.5 ± 2.53	0.75
APOE4 carriers, n (%)	82 (26.5)	59 (29.6)	0.43
Females, n (%)	185 (59.7)	80 (40.2)	< 0.001
Hypertension, n (%)	97 (31.3)	79 (39.7)	0.052
Diabetes, n (%)	13 (4)	12 (6)	0.35
MMSE	29.1 ± 1.1	29 ± 1.1	0.51
MCI (n = 985)			
Sample size, n	586	399	/
Age, years	72.4 ± 8.03	73.7 ± 6.9	0.005
Education, years	15.9 ± 2.87	16.2 ± 2.67	0.08
APOE4 carriers, n (%)	297 (50.7)	204 (48.9)	0.6
Females, n (%)	268 (45.7)	133 (33.3)	< 0.001
Hypertension, n (%)	192 (32.8)	169 (42.4)	< 0.001
Diabetes, n (%)	36 (6.1)	34 (8.5)	0.15
The use of AD medications ^a^, n (%)	161 (27.5)	118 (29.6)	0.47
MMSE	27.6 ± 1.82	27.6 ± 1.86	0.9
AD (n = 372)			
Sample size, n	231	141	
Age, years	74.3 ± 8.22	75.5 ± 7.4	0.15
Education, years	15.1 ± 3.05	15.4 ± 2.7	0.31
APOE4 carriers, n (%)	152 (65.9)	97 (68.8)	0.55
Females, n (%)	113 (48.9)	51 (36.2)	0.02
Hypertension, n (%)	95 (41.1)	50 (35.5)	0.28
Diabetes, n (%)	13 (5.6)	14 (9.9)	0.12
The use of AD medications ^a^, n (%)	177 (76.6)	105 (74.5)	0.64
MMSE	23.2 ± 2.1	23 ± 2.2	0.5

^a^ A subject was regarded as using AD medication if he or she received one of these AD medications, such as Donepezil, Memantine, Galantamine and Rivastigmine.

Abbreviations: CN: normal cognition; MCI: mild cognitive impairment; AD: Alzheimer’s disease; MMSE: mini-mental state examination.

### Cognitive outcome

Mini-mental state examination (MMSE) [16] was used as the cognitive outcome in both cross-sectional analysis and longitudinal analysis. At the screening visit, MMSE was also one of the criteria for the assignment of clinical status. For example, AD patients must have a MMSE score of 24 or lower. Subjects with normal cognition and MCI had a MMSE score between 24 and 30.

### Statistical analyses

Analyses were conducted in each diagnostic group (NC, MCI and AD). Independent t-tests were used to examine the mean differences for continuous variables between non-aspirin users and aspirin users. Chi-square tests were utilized to examine the distribution differences for categorical variables between non-aspirin users and aspirin users. In the longitudinal analysis, linear mixed models were conducted in order to examine the effect of aspirin using conditions on cognitive decline over time using R package “lme4” [17]. Mixed models were conducted within the whole sample and each diagnostic group. Predictors included age, education, gender, APOE4 genotype, hypertension, diabetes, aspirin using status (non-aspirin users vs aspirin users) and their interactions with time. A random intercept for each subject was included in the linear mixed models.

## Results

### Demographic characteristics

At the baseline, this study included 1866 subjects, including 509 subjects with NC, 985 subjects with MCI, and 372 patients with AD. Table 1 demonstrates comparisons of demographic and clinical variables between non-aspirin users and aspirin users within each diagnostic group. Table 2 displays numbers of participants at each visit during which MMSE was examined.

**Table 2:** Numbers of participants at each visit.

	NC		MCI		AD	
Follow-up visits	Non-aspirin users	Aspirin users	Non-aspirin users	Aspirin users	Non-aspirin users	Aspirin users
Baseline	310	199	586	399	231	141
1 y	246	160	510	362	176	112
2 y	230	149	407	283	106	65
3 y	139	78	348	228	5	5
4 y	137	98	238	168	1	1
5 y	86	48	157	105	1	1
6 y	109	79	128	88	0	2
7 y	85	54	97	78	/	/
8 y	68	46	69	49	/	/
9 y	43	16	33	26	/	/
10 y	29	17	17	11	/	/
11 y	26	12	8	9	/	/
12 y	20	8	7	7	/	/
13 y	22	9	3	2	/	/
14 y	5	3	1	0	/	/

Abbreviations: CN: normal cognition; MCI: mild cognitive impairment; AD: Alzheimer’s disease.

In the NC group (n = 509), there were 310 non-aspirin users and 199 aspirin users, respectively. Women were less likely to use aspirin than men (p < 0.001). No significant differences in other variables (age, education, APOE4 genotype, hypertension, diabetes or MMSE) between two groups were found (all p > 0.05).

In the MCI group (n = 985), there were 586 non-aspirin users and 399 aspirin users, respectively. Women were less likely to use aspirin than men (p < 0.001). Aspirin users were older than non-aspirin users. Compared to non-aspirin users, aspirin user had higher percentage of hypertension (p < 0.001). No significant differences in other variables (education, APOE4 genotype, diabetes, the use of AD medications or MMSE) between two groups were found (all p > 0.05).

In the AD group (n = 372), there were 231 non-aspirin users and 141 aspirin users, respectively. Women were less likely to use aspirin than men (p = 0.02). No significant differences in other variables (age, education, APOE4 genotype, hypertension, diabetes, the use of AD medications or MMSE) between two groups were found (all p > 0.05).

#### 1.1. Baseline MMSE scores by aspirin using conditions within each diagnostic group

**Fig 1:**
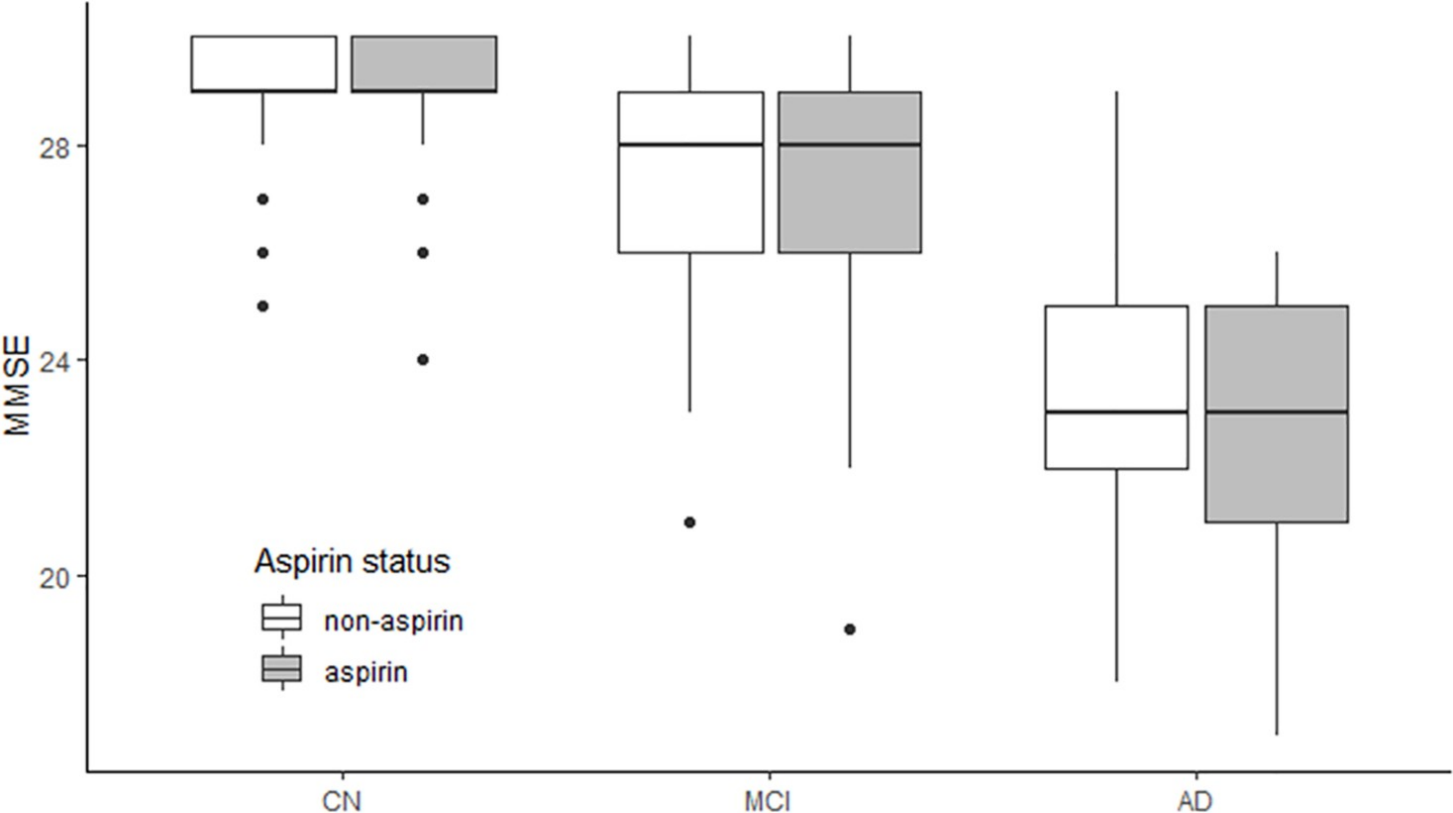
Baseline MMSE scores by aspirin using conditions within each diagnostic group. We did not find a significant difference in MMSE scores between two groups at baseline in any diagnostic group (CN, MCI and AD). Abbreviations: CN: cognitively normal; MCI: mild cognitive impairment; AD: Alzheimer’s disease; MMSE: mini-mental state examination.

As shown in Table 1 and Fig 1, we did not find a significant difference in MMSE scores between two groups at baseline in any diagnostic group (NC, MCI or AD).

#### 1.2. Association of baseline aspirin using conditions with change in MMSE scores over time

**Fig 2:**
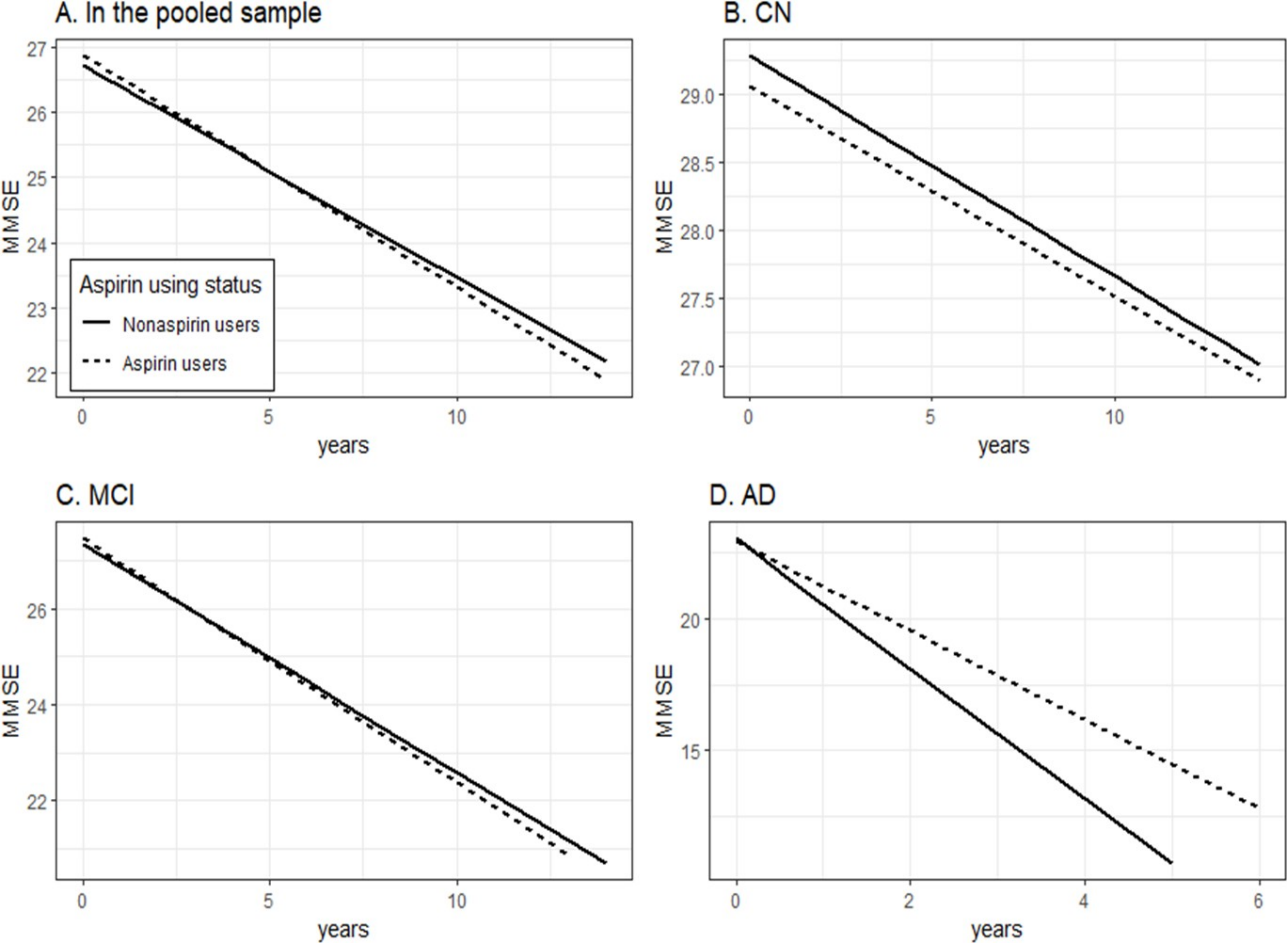
Association of baseline aspirin using conditions with change in MMSE scores over time. Compared to non-aspirin users, aspirin users showed a significantly slower decline in MMSE scores in the AD group (coefficient: 0.7004; p = 0.0024). However, we did find significant differences in changes in MMSE over time between two groups in the whole sample, CN or MCI group (all p > 0.05). Abbreviations: CN: cognitively normal; MCI: mild cognitive impairment; AD: Alzheimer’s disease; MMSE: mini-mental state examination.

To examine the effect of aspirin using conditions (Non-aspirin users vs aspirin users) on change in MMSE scores over time, linear mixed models were conducted within the whole sample and within each diagnostic group (NC, MCI, and AD) (Table 3 and Fig 2). As shown in Table 3 and Fig 2, compared to non-aspirin users, aspirin users showed a significantly slower decline in MMSE scores in the AD group (coefficient: 0.7004; p = 0.0024). However, we did find significant differences in changes in MMSE over time between two groups in the whole sample, NC or MCI group (all p > 0.05).

**Table 3:** Summary of linear mixed models.

	Dependent variable: MMSE		
	Coefficient	SE	P value
Variable (In the whole sample)			
Aspirin users (baseline)	0.1813	0.1704	0.2874
Aspirin users × time	-0.0069	0.0228	0.7626
Variable (CN)			
Aspirin users (baseline)	-0.1346	0.1157	0.2445
Aspirin users × time	0.0088	0.0226	0.6988
Variable (MCI)			
Aspirin users (baseline)	0.0966	0.1863	0.6039
Aspirin users × time	-0.0410	0.0320	0.2003
Variable (AD)			
Aspirin users (baseline)	-0.1363	0.3926	0.7285
Aspirin users × time	0.7004	0.2309	0.0024

Abbreviations: CN: cognitively normal; MCI: mild cognitive impairment; AD: Alzheimer’s disease; MMSE: mini-mental state examination. Notes: Mixed models were conducted within the whole sample and each diagnostic group. Predictors included age, education, gender, APOE4 genotype, hypertension, diabetes, aspirin using status (non-aspirin users vs aspirin users) and their interactions with time.

## Supplementary analysis

In order to examine whether the association of aspirin use with cognitive decline in AD group can be modified by age, APOE4 genotype and sex, three linear mixed-effects models were additionally fitted. The first model included the age*aspirin using status (non-aspirin users vs aspirin users) interaction term, education, gender, APOE4 genotype, hypertension, diabetes, and their interactions with time. The second model included the APOE4 genotype*aspirin using status interaction term, age, education, gender, hypertension, diabetes, and their interactions with time. The third model included the gender*aspirin using status interaction term, age, education, APOE4 genotype, hypertension, diabetes, and their interactions with time. All models included a random intercept for each subject. In the first model, the age*aspirin using status interaction term was not significant (coefficient: 0.0406; p = 0.21). Similarly, in the second model, the APOE4 genotype*aspirin using status interaction term was not significant (coefficient: 0.4019; p = 0.43). However, in the third model, we found that the gender*aspirin using status interaction term was significant (coefficient: -1.0417; p = 0.026), indicating that gender may modify the association of aspirin use with cognitive decline in AD group. Therefore, we further performed a gender-stratified analysis. We found that aspirin use was associated with slower cognitive decline in male AD patients (coefficient: 1.1407; p < 0.001), but not in female AD patients (coefficient: 0.0598; p = 0.8717).

## Discussion

In the cross-sectional analysis, there were no significant differences in MMSE scores between non-aspirin users and aspirin users in subjects with NC, subjects with MCI or patients with AD. In the longitudinal analysis, we detected an association of aspirin use with cognitive decline (MMSE) over time in patients with AD, but not in the NC group or MCI group. Specifically, in AD patients, the use of aspirin at baseline was associated with slower cognitive decline over time.

Previous epidemiological studies suggesting that the use of NSAIDs or aspirin was associated with a decreased risk of developing AD contributed to the hypothesis that alleviation of inflammation in the brain may serve as a new target for slowing down the development of AD [6, 18]. For instance, a previous meta-analysis found that the use of NSAIDs was associated with a decreased risk of the development of AD dementia[19]. In line with this finding, among subjects without dementia, Waldstein and colleagues found that NSAIDs use was associated with slower decline on several cognitive domains [11]. However, another study using the Epidemiology of Hearing Loss Study of participants suggested that the use of aspirin was not associated with progression to cognitive deficits (MCI or dementia diagnosis) [20], which was consistent with other previous studies [14, 21–26]. One potential explanation of these inconsistencies is that this association may be modified by the clinical stages. Therefore, in the present study, we examined the association of aspirin use with cognitive decline in three different diagnostic groups separately. Our findings showed an association of the baseline use of aspirin with cognitive decline (MMSE) over time in patients with AD, but not in the NC group or MCI group.

There are several potential mechanisms by which aspirin or NSAIDs could affect the development of AD. NSAIDs may affect the inflammatory response in AD by the upregulation of the peroxisome proliferator γ (PPAR γ) nuclear transcription factor and by the downregulation of cyclooxygenase-1 and cyclooxygenase-2 [27–29]. In addition, previous animal studies suggested that aspirin could reduce oxidative stress and neuroinflammation in the brain [30, 31]. In transgenic AD mice, inhibiting cyclooxygenase-1 could decrease the levels of amyloid pathologies and improve cognitive performance [32].

There are several potential limitations in the present study. First, given that the sample size of the AD group was relatively small, it would be important for future investigations with larger sample sizes to validate whether the association of the use of aspirin with cognitive decline is dependent on the clinical stages. Second, subjects from the ADNI study represent a convenience sample who are highly educated. Therefore, this may limit the generalizability of our findings. Third, the data on aspirin using conditions were collected based on self-reporting, which may lead to potential bias. Fourth, life style differences between two groups (non-aspirin vs aspirin) might have influenced the findings in the present study. However, the ADNI study did not include these variables, such as levels of physical activities and dietary patterns. Future studies will be needed to address these research questions. Finally, it is important to assess the association of aspirin with the development of other cerebrovascular diseases (e.g. stroke). This notion should be tested in future studies.

In conclusion, we detected an association of the baseline use of aspirin with cognitive decline (MMSE) over time in patients with AD, but not in the NC group or MCI group.
